# Challenging the Paradigm: Long-Term Outcomes in Dialysis-Dependent Patients Undergoing CABG

**DOI:** 10.3390/jcdd12090356

**Published:** 2025-09-16

**Authors:** Ezin Deniz, Tonita Brunkhorst, Florian Helms, Jasmin Hanke, Ali Merzah, Sadeq Ali-Hasan Al-Saegh, Alina Zubarevich, Felix Fleissner, Issam Ismail, Gregor Warnecke, Günes Dogan, Jan Dieter Schmitto, Bastian Schmack, Alexander Weymann, Arjang Ruhparwar, Aron-Frederik Popov

**Affiliations:** 1 Department of Cardiothoracic, Transplant and Vascular Surgery, Hannover Medical School, 30625 Hannover, Germanyschmitto.jan@mh-hannover.de (J.D.S.);; 2 Cardiac Surgery, University Hospital Oldenburg, 36133 Oldenburg, Germany; 3 Cardiovascular Surgery, University Hospital of Kiel, 24118 Kiel, Germany

**Keywords:** CABG, dialysis, cardiac surgery, paradigm, risk score

## Abstract

Dialysis-dependent (DD) patients undergoing coronary artery bypass grafting (CABG) remain a particularly high-risk population with impaired outcomes despite advances in surgical techniques. In this single-center, retrospective cohort study, 97 DD patients (2010–2015) were compared with 488 non-dialysis-dependent (NDD) controls. The primary endpoint was all-cause mortality; the secondary endpoint was major adverse cardiac and cerebrovascular events (MACCE). Median follow-up was 5.4 ± 2.1 years. DD patients had significantly higher perioperative mortality (10.3% vs. 3.1%, *p* = 0.002) and markedly reduced overall survival (OS) (40.8% vs. 82.1% at 5 years). Dialysis dependence conferred an 8.4-fold increase in mortality risk and a 2.6-fold increase in MACCE risk. Increasing age, diabetes, and critical preoperative state were independent predictors of an adverse long-term outcome. While arterial grafting improved survival in NDD patients, no comparable benefit was observed in DD patients, possibly due to vascular calcification, limited conduit availability, and reduced graft patency. EuroSCORE II adequately predicted perioperative mortality (AUC = 0.78 in DD patients) but demonstrated poor discriminatory power for long-term survival (AUC = 0.67 at 5 years). These findings highlight the urgent need for dialysis-specific risk models. Despite poor long-term prognosis, DD patients with low-risk EuroSCORE II profiles experienced the most relative benefit from CABG.

## 1. Introduction

Since the introduction of coronary artery bypass graft surgery over 50 years ago, the results have, with constant optimization of surgical techniques, improved considerably [1]. Nevertheless, the long-term mortality and morbidity for high-risk patients such as patients with diabetes, reduced left ventricular function (LVEF), chronic lung disease (CLD), impaired renal function and multi-morbidity, as well as elderly patients, have remained impaired [2]. Patients with kidney insufficiency requiring dialysis, which are the focus of this study, also belong to the high-risk group [3,4,5,6].

The number of patients in need of dialysis therapy is rising worldwide [5]. Estimations suggest numbers will rise from 100.202 in 2017 to 120.000–123.000 in 2040 in Germany alone [7]. This is mainly accounted for by higher life expectancy, diabetes, and hypertension [8]. These patients bear a high risk for cardiac artery disease (CAD) and subsequent morbidity and mortality [9,10]. Studies in the past have shown the superiority of CABG over PCI regarding the long-term outcome for dialysis-dependent patients in need of therapy [11]. Yet, the early- and long-term mortality and morbidity rates after surgery remain high [3,4,5,6]. Among other issues, the poor outcome is associated with renal impairment being an independent risk factor [6,12,13,14], but also dialysis-dependent patients holding more comorbidities [3,4,15].This urges on the search for characteristics in favor of survival and suitable survival predictors.

The European System for Cardiac Operative Risk Evaluation (EuroSCORE; euroscore.org) is a well-known predictor for perioperative mortality after cardiac surgery [16]. Long-term predictors are still lacking. There have only been a few attempts to apply the EuroSCORE as a long-term predictor, with diverging conclusions [12,17,18,19].

Our study sought to investigate the outcome of dialysis-dependent patients undergoing CABG alongside a control group of non-dialysis-dependent patients. The aim was to detect characteristics that are detrimental regarding the patients’ long-term survival and compare the outcome with that of other risk groups. Additionally, the EuroSCORE II was tried as a predictor for long-term survival.

## 2. Methods

### 2.1. Patients and Data Collection

This was a single-center, retrospective study at the University Hospital Hannover. Data from 97 dialysis-dependent patients from 2010 to 2015 were collected, and a control group of 488 consecutive non-dialysis-dependent patients was created; the group underwent CABG. The ratio of cohorts was set at 1:5 for credible study power [20]. The DD cohort included patients with chronic kidney failure in need of hemodialysis prior to surgical intervention, irrespective of their creatinine level. Patients with acute renal failure were excluded from this study. Elderly patients were defined as 70 years and older. Preoperative data, surgical details, the perioperative outcome, and complications were obtained from electronical hospital records. Follow-up details were acquired from the patients, family members, their physicians, and cardiologists. Ultimately, if no information could be obtained, the Citizens Registration Office was contacted for information on their vital status. 

### 2.2. Long-Term Survival

The primary endpoint in our study was defined as all-cause death, and the secondary endpoint was defined as major adverse cardiac and cerebrovascular events (MACCE; including myocardial infarction, PCI, re-bypass surgery and stroke) or death, irrespective of cause.

### 2.3. Statistical Analysis

All statistical analyses were performed with IBM SPSS Statistics Version 25.0.

Baseline characteristics were presented as mean ± deviation or percentages. Differences between the NDD and DD cohort were compared with Chi-Square-Test for categorial variables and the Mann–Whitney-U-Test for continuous variables as they tended to be unevenly distributed.

Overall survival and freedom of MACCE were analyzed and visualized with the Kaplan–Meier Method. Differences were assessed using the log-rank test.

Potential risk factors such as preoperative and surgical characteristics were set into relation with late mortality using Cox-Proportional-Hazard-Analysis. Univariate analysis was performed. Variables with significance were then implemented into multivariate analysis. Multivariable Cox regression analysis was performed to adjust for baseline imbalances. Propensity score methods, such as matching or inverse probability weighting, were considered, but given the very limited number of dialysis-dependent patients, these approaches were not feasible without substantial loss of statistical power.

Kaplan–Meier curves were further used to compare OS among different risk groups. Therefore, the EuroSCORE II was stratified into groups of low-risk (<5%), medium-risk (5–10%), and high-risk (>10%).

For the evaluation of the EuroSCORE II as a mortality predictor, we utilized Kaplan–Meier curves and the ROC curve. The AUC was interpreted as follows: 0.9–1.0 = excellent; 0.8–0.9 = good; 0.7–0.8 = fair; 0.6–0.7 = poor; and 0.5–0.6 = fail.

Results were considered statistically significant by a *p*-value of ≤0.05 with a confidence interval (CI) of 95%. In case of incomplete follow-up, the case was excluded from further outcome analysis.

## 3. Results

All 97 dialysis-dependent patients undergoing CABG from 2010 to 2015 were included, as were 488 non-dialysis-dependent patients in the control group. However, six NDD patients had to be excluded from the outcome analysis, due to a lack of follow-up information after discharge.

### 3.1. Baseline Characteristics

Preoperative demographics and preclinical conditions are depicted in Table 1. The average age was 67.9 ± 9.8 years for NDD and 66.9 ± 9.9 years for DD patients (*p* = 0.362). The DD group had a higher share of male patients (NDD = 75.8%; DD = 79.4%; *p* = 0.045). There were no significant differences in priority of surgery with a distribution of elective (NDD = 58.6%; DD = 56.7%; *p* = 0.728), urgent (NDD = 32.4%; DD = 34.0%; *p* = 0.753), emergent (NDD = 8.8%; DD = 9.3%; *p* = 0.883), and salvage (NDD = 0.2%; DD = 0.0%; *p* = 0.655). DD patients showed a significantly higher count for diabetes (NDD = 28.3%; DD = 43.3%; *p* = 0.003), peripheral artery disease (PAD) (NDD = 19.3%; DD = 45.4%; *p* = 0.000), CLD (NDD = 10.2%; DD = 17.5%; *p* = 0.040), recent cardiac infarction (NDD = 21.9%; DD = 36.1%; *p* = 0.003), neurological dysfunction, e.g., stroke or transient ischemic attack (NDD = 1.6%; DD = 14.4%; *p* = 0.000), and a higher EuroSCORE II (NDD = 4.4% ± 7.4; DD = 9.8% ± 9.8; *p* = 0.000). However, the NDD cohort presented a significantly higher share of hyperlipidaemia (NDD = 64.5%; DD = 44.3%; *p* = 0.000).

### 3.2. Surgical Details

The intraoperative data are summarized in Table 2. DD patients were involved in more concomitant surgeries, especially surgeries including two additional procedures despite the CABG (NDD = 8.4%; DD = 16.5%; *p* = 0.014). Prominent in our study is the difference in revascularization-methods between both groups. NDD patients obtained significantly more arterial bypasses including total arterial revascularization (TAR) (NDD = 23.0%; DD = 0.0%; *p* = 0.000), left internal thoracic artery bypasses (LITA) (NDD = 84.4%; DD = 42.3%; *p* = 0.000), and A. radialis (NDD = 21.6%; DD = 2.1%; *p* = 0.000) bypasses, whereas DD patients received more venous bypasses (NDD = 65.8%; DD = 100.0%; *p* = 0.000). The type and number of revascularized coronaries revealed no significant differences. An off-pump coronary artery bypass (OPCAB) was received by 3.1% of NDD patients and none were received by DD patients.

### 3.3. Overall Survival

Our mean follow-up was 5.4 ± 2.1 years (maximum: 7.7 years). The short-term mortality was significantly higher for DD patients with a perioperative death rate of 10.3% compared to NDD patients (3.1%; *p* = 0.00). The OS rate for DD compared to NDD patients was 73.2% ± 4.5 to 92.5% ± 1.2 in year 1, 54.4% ± 5.2 to 87.1% ± 1.5 in year 3, and 40.8% ± 5.3 to 82.1% ± 1.7 in year 5. The rate of MACCE free patients was 70.1% ± 4.6 to 89.6% ± 1.4 within the first year, 47.2% ± 5.3 to 80.9% ± 1.8 within the third year, and 29.5% ± 5.0 to 72.8% ± 2.0 within the fifth year.

**Figure 1:**
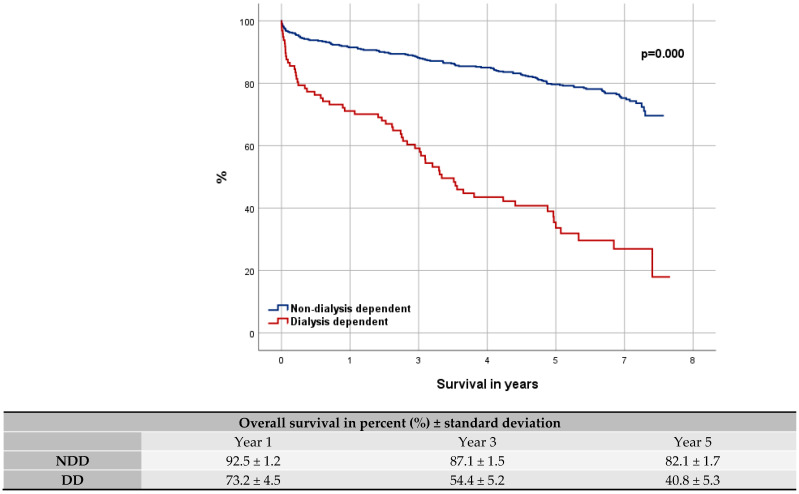
Kaplan–Meier curve: overall survival.

**Figure 2:**
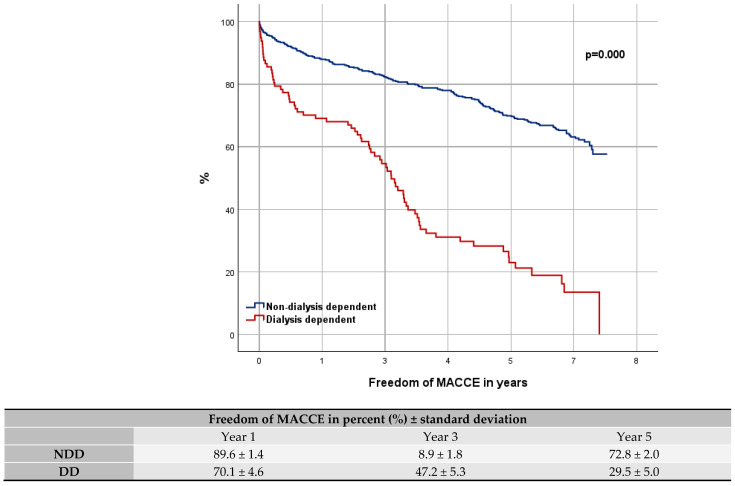
Kaplan–Meier curve: freedom of major adverse cardiac and cerebrovascular events.

The Kaplan–Meier curves show the OS (log-rank *p* = 0.000; Figure 1) and the MACCE free trend (log-rank *p* = 0.000; Figure 2) between NDD and DD cohorts. For analysis the Cox-Proportional-Hazard-Analysis was used, revealing an over-four-times higher risk of DD patients for mortality [*p* = 0.000; HR = 4.779 (95% CI 3.424–6.670)] and MACCE [*p* = 0.000; HR = 4.089 (95% CI 3.037–5.504)].

### 3.4. Comparison with Elderly and High-Risk Patients

**Figure 3:**
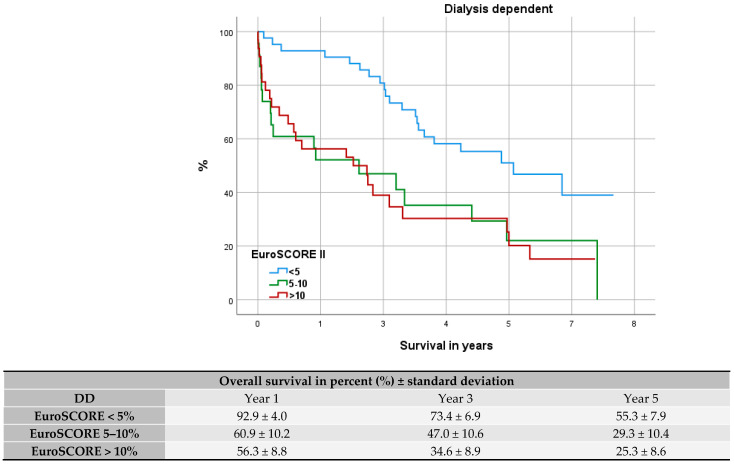
Kaplan–Meier curve: survival of dialysis-dependent patients within EuroSCORE II groups.

**Figure 4:**
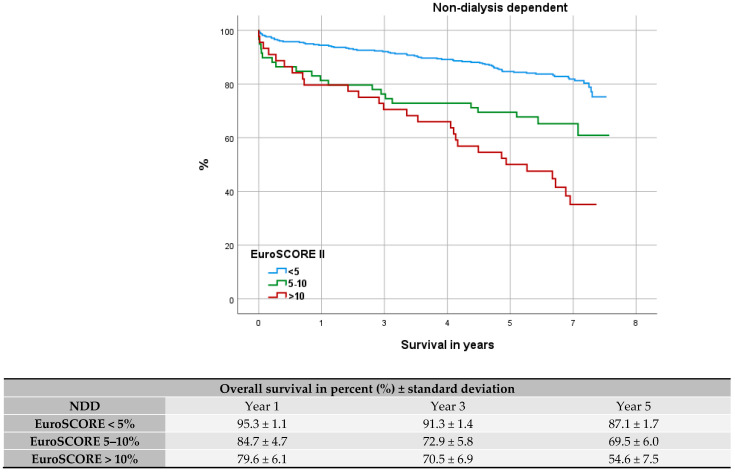
Kaplan–Meier curve: survival of non-dialysis-dependent patients within EuroSCORE II groups.

**Figure 5:**
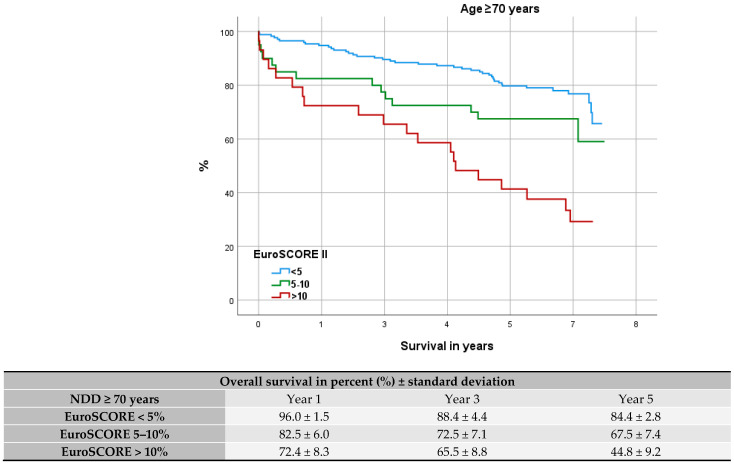
Kaplan–Meier curve: elderly patients (age ≥ 70 years) within EuroSCORE II groups.

After stratifying our cohorts into 3 EuroSCORE II groups (<5%, 5–10%, >10%), we further visualized their survival trend via Kaplan–Meier curves for DD, NDD, and elderly patients (see Figure 3, Figure 4 and Figure 5). Median survival among DD patients was low-risk (EUROSCORE II < 5%): 5.6 years (CI 95% = 3.3–7.9), medium-risk (EUROSCORE II 5–10%): 2.2 years (CI 95% = 0.0–4.6), and high-risk: (EUROSCORE II > 10%) 2.1 years (CI 95% = 3.3–8.4). In every risk group the DD cohort showed a significantly worse OS (EUROSCORE II 5%: *p* < 0.001; EUROSCORE II 5–10%: *p* < 0.001; ES > 10%: *p* = 0.004). When compared the Kaplan–Meier curves, low-risk DD patients had an OS comparable to NDD patients with a high-risk profile [median survival: 5.8 years (CI 95% = 3.3–8.4)]. DD patients with a medium-to-high-risk profile (EUROSCORE II > 5%) presented a worse outcome than elderly patients with high-risk profile (EUROSCORE II > 10%) [median survival: 4.3 years (CI 95% = 3.2–5.3)] (see Figure 5). The respective OS after 1, 3, and 5 years is tabulated under the Kaplan–Meier curves.

### 3.5. Uni- and Multivariate Analysis

Uni- and multivariate statistics are shown in Table 3 and Table 4. A total of 17 potential risk factors were included. Of these, including all patients, dialysis dependency, increasing age, decreased left ventricular ejection fraction (LVEF), diabetes, peripheral artery disease (PAD), neurological Dysfunction (ND), e.g., stroke or transient ischemic attack, chronic lung disease (CLD), aorta surgery and CABG with venous grafts had a significant negative impact on the OS. Of these the dialysis dependency was the most powerful coefficient (HR = 3.134; 95% CI 2.143–4.588).

With focus on the dialysis-dependent patients only, increasing age, diabetes, and, furthermore, the critical preoperative state as the strongest variable (HR = 5.016; 95% CI 1.383–18.193) were independent risk factors for a reduced long-term survival.

### 3.6. Significance in Choice of Graft Material

**Figure 6:**
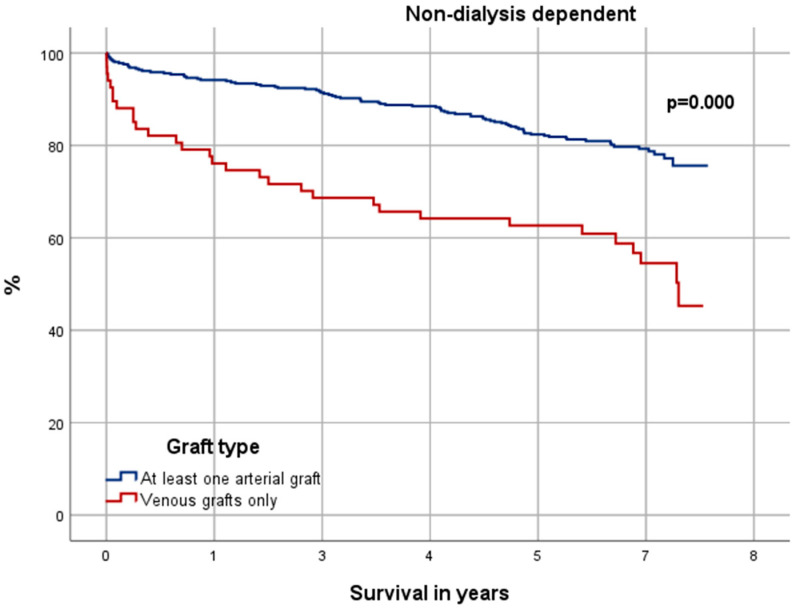
Kaplan–Meier curve: non-dialysis-dependent patients with venous grafts only vs. at least one arterial graft.

**Figure 7:**
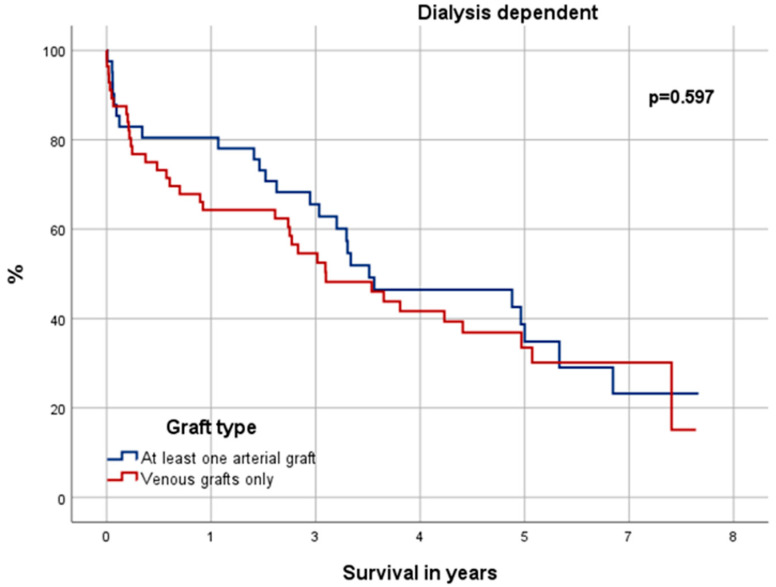
Kaplan–Meier curve: dialysis-dependent patients with venous grafts only vs. at least one arterial graft.

Receiving at least one arterial graft instead of venous grafts only had a significant positive effect on the OS for NDD patients (*p* = 0.000) (see Figure 6). Yet there was neither significant difference for the DD cohort in total (*p* = 0.597) (see Figure 7) nor within its risk groups [low-risk (*p* = 0.984); medium-risk (*p* = 0.984); high-risk (*p* = 0.920)] (not depicted). The Cox-Proportional-Hazard-Analysis (see Table 3 and Table 4) revealed that venous grafts only had a significant negative impact on the OS of NDD patients [HR = 1.47 (CI 95% = 1.01–2.13), *p* = 0.034], but not for DD patients.

### 3.7. Evaluation of Fit: EuroSCORE II

The EuroSCORE II revealed a significantly higher risk score for DD patients (DD = 9.8% ± 9.8; NDD = 4.4% ± 7.4; *p* = 0.000) (see Table 1). The actual perioperative mortality rate was 10.3% for DD and 3.1% for NDD patients (see Table 2); thus, this was comparable to the predicted EuroSCORE II.

**Figure 8:**
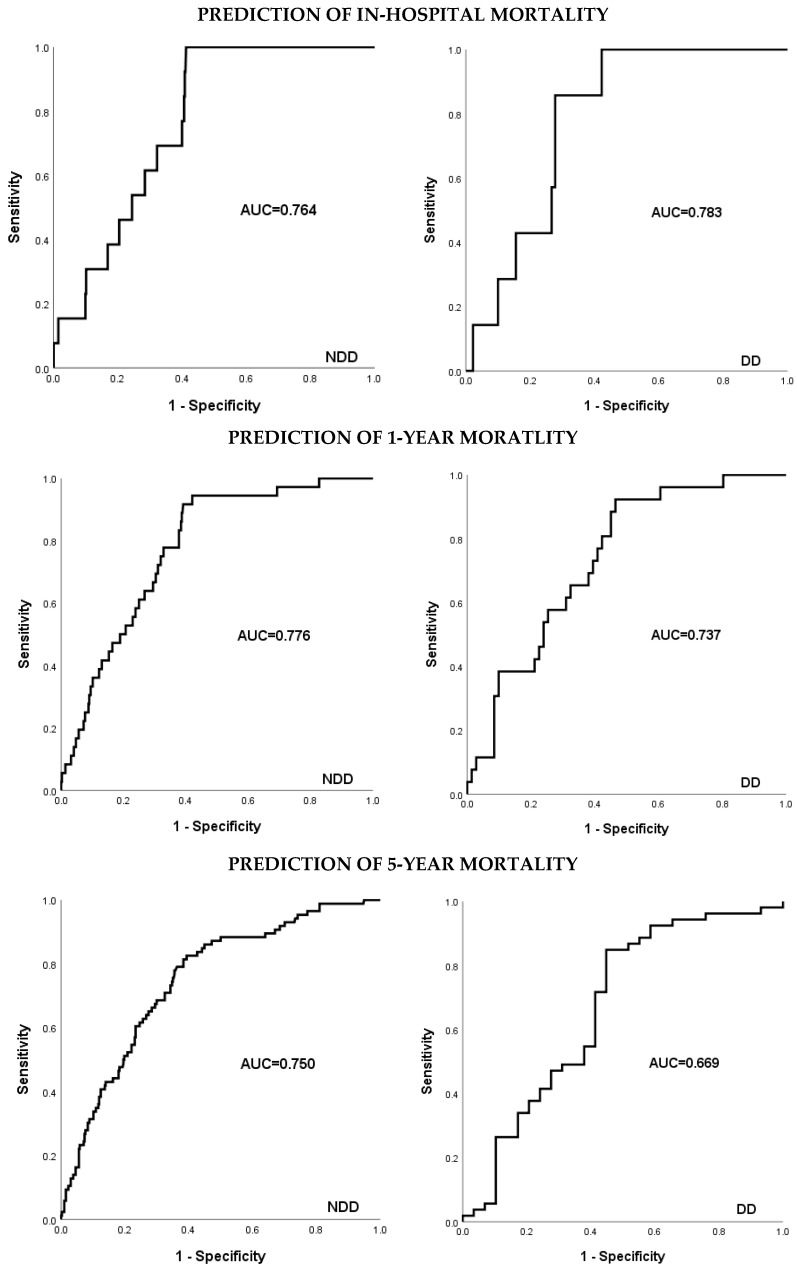
ROC curves for the EuroSCORE II over time. ROC = Receiver Operating Characteristic; AUC = area under the curve; NDD = non-dialysis-dependent; DD = dialysis-dependent.

According to the area under the Receiver Operating Characteristic (ROC) curve the EuroSCORE II proved a fair fit for perioperative mortality prediction (NDD: AUC = 0.764; DD: AUC = 0.783) and after 1 year (NDD: AUC = 0.776; DD: AUC = 0.737). The value of fit disperses though to AUC = 0.750 and AUC = 0.673 for NDD and DD patients after 5 years. The value as predictor for DD patients at this point was only poor (see Figure 8). The Kaplan–Meier curves (see Figure 3 and Figure 4) depict the survival trend for each cohort stratified into three risk groups. Here the higher risk profiles show crossovers, especially among DD patients, and therefore a proportional hazard is not given.

## 4. Discussion

### 4.1. Overall Survival

Our analysis found that the death and MACCE rates were 40.8% ± 5.3 and 29.5% ± 5.0 within five years for DD patients, and thus they display an 8.4-fold increase in mortality risk and 2.6-fold increase in MACCE risk compared to the NDD group. Former studies repeatedly stated poor early- and long-term survival for CABG among DD patients. The mortality rate within 1 year and 5 years ranged between 20 and 28% and 38 and 51%, respectively [3,4,5,6,13,15]. In our study the survival rates were 73.2%, 54.4% and 40.8% after 1 year, 3 years, and 5 years.

Both in this study and in previous studies, renal insufficiency with dialysis dependency was found to be an independent risk for OS [12,13,14]. Dialysis dependency connotes poor outcome prospects regardless of CABG [21]. Krämer et al. for example observed a 50% survival after 5 years of dialysis dependency [22]. Also, for this outcome, unevenly distributed risk factors must be taken into consideration, as DD patients present more comorbidities [3,4,15].

In order to dissolve the disproportion of risk factors, we stratified the cohorts into three risk groups based on the patients’ EuroSCORE II. In all risk groups the DD cohort showed a significantly worse long-term outcome compared to the NDD cohort. Furthermore, DD patients with a higher risk profile (EUROSCORE II > 5% presented with markedly reduced OS, as it was reduced by more than half compared to DD patients with low-risk profile. Dacey et al. and others observed in their studies the rising risk for mortality with increasing numbers of comorbidities [4,23].

Despite the markedly impaired OS, 50% of the DD patients with a high-risk profile nevertheless had a survival of at least 2 years after CABG.

### 4.2. Uni- and Multivariate Analysis

Resembling other studies [3,4,5,14,15] our uni- and multivariate analysis revealed dialysis dependency, increasing age, LVEF, PAD, CLD, aorta surgery, and diabetes being independent predictors for long-term mortality. The same could not be concluded for female gender, previous stroke, recent cardiac infarction, previous CABG, or concomitant surgery. Similar to Gao et al. our results reflect that demographics and chronic diseases play a crucial role in the long-term outcome [24].

Additionally, our analysis detected CABG surgery that involved exclusively venous grafts as an independent risk factor, which will be discussed in a later section.

Focus on the DD cohort diabetes and increasing age showed, concurring with former studies, a significant negative impact on the long-term outcome [23,25,26]. Additionally, the critical preoperative state implied a five-times higher risk compared to the non-critical preoperative state. Emergency CABGs are known to have an increased risk for short-term mortality [27,28], yet publications that confirm the correlation between long-term outcome and a critical preoperative state among DD patients are lacking. The results in this study are most likely due to the limited number of cases. Our analyses show that a total of three patients among the DD cohort were in a preoperative critical condition. These died after 29, 61, and 109 days, which could explain the high hazard ratio.

### 4.3. Comparison to High-Risk and Elderly Patients

With growing numbers of patients undergoing CABG, elderly and high-risk patients are increasingly being considered for myocardial revascularization, too.

Former studies have discussed the benefit for patients with high-risk profile undergoing CABG surgery [29,30]. A high-risk profile is considered in the presence of comorbidities such as diabetes, peripheral vascular disease, chronic lung disease, reduced left ventricular function, and renal insufficiency. Both multi and comorbid diseases are associated with high operational and anesthetic risk [29] and often produce poor results leading to significant mortality and morbidity [30]. Our Kaplan–Meier graphs of DD and NDD cohorts, stratified into three EuroSCORE II risk groups, show that DD patients with a low-risk profile have a similar OS tendency compared to an NDD patient with a high-risk profile. After approximately 5.7 years there was a 50% survival rate in both cohorts.

Also, the justifiability of elderly patients undergoing CABG has been discussed in the past as they bear a higher risk for mortality and complications [31]. The mortality and multiorgan morbidity increase significantly with advancing age. When compared, the elderly non-dialysis-dependent patients with a high-risk profile nevertheless had a better outcome than DD patients with a medium-risk profile. The median survival of elderly patients with a high-risk profile was 4.3 years (CI 95% = 3.2–5.3) and was thus twice as long compared to DD patients with a higher-risk profile.

### 4.4. Significance in Choice of Graft Material

DD patients primarily received venous grafts. Though previous studies have shown the superiority of TAR, internal thoracic artery (ITA), and radial artery grafts over venous grafts [30,31,32,33,34], our DD patients received significantly fewer arterial grafts compared to our control group. In fact, none received a TAR. A possible explanation for this is the arteries not being suitable. Hemodialysis-dependent patients are at a higher risk of developing peripheral artery disease due to their high incidence of comorbidities [15]. Also, hemodialysis-dependent patients may be in need of an arteriovenous fistula, which involves the usage of the radial artery, for chronic hemodialysis [31]. This procedure, described by Brescia and Cimino, is the first choice for chronic hemodialysis [35]. In addition, there is the possible occurrence of a subclavian steal phenomenon [11,15].

The LITA may be unavailable due to an enlargement of the left ventricle, difficult harvest, accidental injury during harvest, calcification of the LITA, or a mismatch with the left anterior descending artery (LAD) and LITA [36]. According to Li et al. the unavailability of the LITA graft may lie at 4–9% [36]. In connection with TAR, it was shown that bilateral internal thoracic artery bypasses (BITA) are superior to venous grafts, but should not be performed in patients with a high risk of sternal complications. These risks include patients with a Body Mass Index > 35 kg/m^2^, severe CLD, and uncontrolled diabetes [37]. Both of our cohorts had high incidences of CLD, adipositas, and diabetes, which could explain why only 1% of DD and 2% of NDD patients received a BITA.

These circumstances suggest DD patients received inferior bypasses with lower patency, which consequently affects their outcome negatively. Shilane et al., for example, suggested ITA bypasses to have a 12–15% better patency than venous grafts after 5 years among DD patients [38]. The results of this study, however, could not support that thesis. Our Kaplan–Meier grafts confirm a significantly improved outcome for NDD patients who received at least one arterial graft over NDD patients with venous grafts only. Yet, there was no significant difference among the DD cohort. Even when taking a closer look by stratifying the DD cohort into EuroSCORE II subgroups, no significant improved outcome for DD patients with arterial grafts can be found. We hypothesize that this absence of benefit may be related to accelerated vascular calcification, frequent need for arteriovenous fistulas limiting radial artery availability, and impaired graft patency under chronic hemodialysis conditions. These mechanisms may outweigh the well-established advantages of arterial conduits observed in non-dialysis populations.

According to former studies saphenous grafts have a patency of 81–88% after five years and 50–74% after ten years [32,33,34]. DD patients in our study had a median survival of 3.2 years (CI 95% = 2.48–3.92) and an OS of 40.8% after 5 years. Given an OS that is shorter than the observed patency of the venous grafts, the graft choice could be irrelevant. This is supported by our uni- and multivariate analysis which detected venous grafts only as independent risk factor for long-term mortality among NDD patients but not DD patients.

Furthermore, advantages of the venous graft are not to be neglected, as they are easier to harvest, are more available, have reduced surgery time and have a lower risk for vessel spasms. Further studies, however, will be necessary to confirm whether the choice of graft impacts the outcome of DD patients that undergo CABG.

### 4.5. ONCAB vs. OPCAB

There are two main ways to perform a CABG: off-pump coronary artery bypass surgery (OPCAB) and on-pump coronary artery bypass surgery (ONCAB). In the past years the focus of research appeared to be on OPCAB as this is associated with fewer complications, such as vasoplegic syndrome, an inflammatory response, neurological complications, and a coagulation deficit [39,40]. Nevertheless, it appears surprising that none of the DD patients and only 3.1% of the NDD patients in our study received an OPCAB. Although several studies have reported improved outcomes with off-pump CABG in patients with impaired renal function, all patients in our cohort underwent on-pump CABG. This reflects that at our institution, on-pump CABG remains the standard approach, guided by institutional expertise and protocols. In addition, anatomical constraints such as poor visualization of circumflex territories and lack of suitable radial arteries in DD patients likely contributed to the absence of OPCAB utilization in this cohort [39,40].

Although preoperative conditions such as an impaired nervous system and arteriosclerotic changes in the aorta indicate an OPCAB, they carry the risk of incomplete revascularization in multivessel coronary artery disease, since diagonal and circumflex territories can often only be inadequately visualized [41,42]. Therefore, more than half of the elective patients at the University Hospital Hannover received ONCAB with T-graft revascularization using a LITA and radial artery bypass. For cannulation of the heart–lung machine, clamping of the ascending aorta is still necessary, but when the radial artery bypass is implemented, clamping is not required, which reduces the risk of cerebral complications. Over time, this technology has become a standard procedure at the University School Hannover, and it has been shown to be safe and efficient [41,43,44]. DD patients however are most commonly not suitable for the described procedure. They often lack availability of radial artery grafts due to arteriosclerosis and their previous use for arteriovenous fistula, and have increased risk for postoperative risk for vascular spasms, particularly in association with coronary-subclavian-steal-syndrome [15]. Instead, they predominantly received venous grafts. Only two patients received a T-graft, which was completed with a venous graft.

### 4.6. Evaluation of Fit: EuroSCORE II

The EuroSCORE has been adjusted in the past [16]. Among others, the risks regarding renal impairment and the type of cardiac surgery have been refined, providing more flexibility within the variables [45]. On these grounds we decided to investigate the more recent EuroSCORE II as short- and long-term mortality predictors. Ad N et al. and others validated the EuroSCORE II as having improved risk evaluation for short-term mortality [19,45]. There have only been few attempts though to extend its use as a long-term predictor. Two studies found the additive EuroSCORE to have good predictive value for mid- and long-term survival [17,18].

In our study the EuroSCORE II performed adequately for perioperative prediction but clearly lost predictive accuracy over time, particularly among dialysis-dependent patients (AUC = 0.669 at 5 years). These results underscore the insufficiency of one-size-fits-all models and highlight the urgent need for dialysis-specific or augmented risk algorithms to improve prognostication and support surgical decision-making. Although EuroSCORE II incorporates renal function, our findings indicate that even dialysis-dependent patients with otherwise favorable EuroSCORE II profiles had impaired long-term survival. This underlines that the score showed reasonable performance for perioperative risk prediction but lost predictive utility during long-term follow-up, particularly in dialysis-dependent patients.

Spiliopoulos et al.’s attempt to deploy the EuroSCORE II as a mid-term predictor found the score losing its calibration with higher risk profiles [19]. Similar results can be summarized from our Kaplan–Meier curves as groups with higher-risk profiles were inclined to cross over, especially among the DD group (see Figure 3 and Figure 4). Furthermore, adverse to Da Marie et al., our uni- and multivariate analysis did not confirm the EuroSCORE II as an independent risk factor for long-term outcomes [18].

Overall, we support Barili et al.’s proposition for the EuroSCORE II to be associated with, but not a direct measure for, long-term survival as the performance fades with time. Thus, a different algorithm would be necessary for the risk factors [12]. Moreover, we suggest a stronger weighting for dialysis dependency due to high mortality rates.

### 4.7. Limitations

This is a single-center, retrospective study, which may limit generalizability due to institutional practices and patient-population characteristics. Imbalances in baseline characteristics between DD and NDD cohorts could introduce residual confounding despite stratification. While we adjusted for baseline comorbidities in multivariable models, the limited size of the dialysis-dependent cohort precluded robust propensity score matching or inverse probability of treatment weighting analyses. As such, residual confounding cannot be excluded, and our findings should be interpreted with caution. Therefore the relatively small size of the DD cohort reduces statistical power, particularly for subgroup and multivariate analyses. Data on graft patency and cause-specific mortality were not available, restricting the assessment of long-term graft performance and causes of death. The study focused exclusively on isolated and concomitant CABG procedures The heterogeneity in surgical indications, such as concomitant valve procedures, redo surgery, and endocarditis, may have influenced outcomes. Due to the limited size of the dialysis-dependent cohort, subgroup analyses restricted to isolated CABG patients were not feasible. We attempted to mitigate this limitation through multivariable adjustment, but residual confounding cannot be excluded.

Furthermore, while EuroSCORE II was applied for long-term outcome prediction, it was primarily designed for perioperative risk; the results should therefore be interpreted cautiously. Finally, risk overestimation in small subgroups, such as patients in a critical preoperative state, cannot be excluded. Overall, these findings should be considered preliminary evidence requiring external validation in larger, multicenter datasets.

## 5. Conclusions

Dialysis-dependent patients bear a high risk for long-term mortality and morbidity with an 8.4-fold increase in mortality risk and a 2.6-fold increase in MACCE risk within 5 years after CABG compared to NDD patients. On the one hand, this is associated with the fact that dialysis dependency represents an independent risk factor and on the other hand that DD patients are associated with more comorbidities. After stratifying the cohorts into EuroSCORE II risk groups, the DD patients nevertheless had a significantly worse OS. In this regard advanced age, diabetes, and critical preoperative status were independent risk factors for reduced OS. DD patients with a low-risk profile showed a poorer outcome than NDD and elderly patients with higher-risk profiles. Nevertheless, 50% of DD patients with a high-risk profile have a minimum survival of 2 years after CABG.

Though our DD cohort received significantly fewer arterial grafts, which are considered superior to venous grafts, no significant impact on the outcome among DD patients was detected in our study.

The EuroSCORE II was applicable as a perioperative mortality predictor, also with regards to the DD cohort. Its value as a long-term predictor, however, faded with time, especially among DD patients. Hence, we suggest a different algorithm is required, as well as a greater weighting of dialysis dependency as a coefficient.

Given the long-term results, dialysis patients with a low-risk profile benefit most from CABG surgery.

## Figures and Tables

**Table 1:** Preoperative details.

Variable	NDD n = 488	DD n = 97	*p*-Value
Gender (male)	75.8%	79.4%	0.045
Age	67.9 ± 9.8	66.9 ± 9.9	0.362
Age ≥ 70	49.8%	48.5%	0.809
Systemic hypertension	84.4%	86.6%	0.586
Diabetes	28.3%	43.3%	0.003
Hyperlipidemia	64.5%	44.3%	0.000
PAD	19.3%	45.4%	0.000
BMI > 30 kg/m^2^	31.1%	26.8%	0.396
Previous stroke	4.1%	6.2%	0.362
CLD	10.2%	17.5%	0.040
MI ≤ 90 days	21.9%	36.1%	0.003
ND	1.6%	14.4%	0.000
Active endocarditis	0.4%	2.1%	0.071
AP	19.9%	19.6%	0.949
Critical preoperative state	4.3%	3.1%	0.583
Previous cardiac surgery	3.3%	6.2%	0.169
Previous PCI	23.3%	22.9%	0.903
LVEF			
good (≥50%)	66.0%	50.5%	0.004
moderate (50–31%)	19.3%	19.6%	0.941
poor (30–21%)	8.4%	19.6%	0.001
very poor (≤20%)	6.4%	10.3%	0.163
NYHA			
I	41.0%	13.4%	0.000
II	36.9%	50.5%	0.012
III	18.9%	30.9%	0.008
IV	3.3%	5.2%	0.364
PHT			
good	93.2%	82.5%	0.001
moderate	6.1%	12.4%	0.030
severe	0.6%	5.2%	0.000
Priorit y			
elective	58.6%	56.7%	0.728
urgent	32.4%	34.0%	0.753
emergency	8.8%	9.3%	0.883
salvage	0.2%	0%	0.655
EuroSCORE II	4.4 ± 7.4	9.8 ± 9.8	0.000

NDD = non-dialysis-dependent; DD = dialysis-dependent; PAD = peripheral arterial disease; BMI = Body Mass Index; CLD = chronic lung disease; MI = myocardial infarction; ND = neurological dysfunction (e.g., stroke or transient ischemic attack); AP = angina pectoralis (chest pain); PCI = percutaneous coronary intervention; NYHA = New Yorker Heart Association; PHT = pulmonary hypertension; EuroSCORE = European System for Cardiac Operative Risk Evaluation.

**Table 2:** Surgical details.

Variable	NDD n = 488	DD n = 97	*p*-Value
Surger y weight			
Isolated CABG	69.9%	59.8%	0.051
2 Procedures	21.7%	23.7%	0.666
3 Procedures	8.4%	16.5%	0.014
Aorta surgery	5.3%	6.2%	0.734
Surgery duration (min)	207.3 ± 67.1	216.8 ± 69.1	0.220
Bypass duration (min)	98.7 ± 44.7	114.3 ± 52.7	0.001
Clamp time (min)	54.7 ± 28.5	56.9 ± 36.3	0.843
Number of anastomosis	3.8 ± 1.3	3.8 ± 1.3	0.475
OPCAB	3.1%	0.0%	0.206
Grafts			
LITA	84.4%	42.3%	0.000
RITA	3.7%	1.0%	0.173
BITA	2.0%	1.0%	0.500
A. radialis	21.6%	2.1%	0.000
TAR	23.0%	0.0%	0.000
Venous	65.8%	100.0%	0.000
Venous only	13.9%	57.7%	0.000
LAD	91.9%	85.6%	0.048
RCX	73.9%	66.0%	0.113
RCA	67.2%	69.1%	0.722
Vascularization of			
1 Coronary artery	15.6%	19.6%	0.328
2 Coronary arteries	35.0%	40.2%	0.333
3 Coronary arteries	48.2%	40.2%	0.152

NDD = non-dialysis-dependent; DD = dialysis-dependent; CABG = coronary arterial bypass graft; min = minutes; OPCAB = off-pump coronary artery bypass; LITA = left internal thoracic artery; RITA = right internal thoracic artery; BITA = bilateral internal thoracic artery; TAR = total arterial revascularization; LAD = left anterior descending artery; RCX = ramus circumflexus; RCA = right coronary artery.

**Table 3:** Cox-Proportional-Hazard-Analysis for all patients.

	Univariate		Multivariate	
	Hazard Ratio (95% CI)	*p*-Value	Hazard Ratio (95% CI)	*p*-Value
Dialysis dependency	4.464 (3.260–6.113)	0.000	3.110 (2.148–4.513)	0.000
Gender (male)	0.718 (0.527–1.008)	0.056		
Age	1.046 (1.029–1.064)	0.000	1.057 (1.037–1.077)	0.000
LVEF	0.975 (0.965–0.984)	0.000	0.980 (0.968–0.992)	0.001
Hypertension	1.270 (0.820–1.968)	0.284		
Diabetes	1.765 (1.313–2.373)	0.000	1.560 (1.143–2.129)	0.005
PAD	2.407 (1.779–3.257)	0.000	1.646 (1.182–2.291)	0.003
CLD	2.598 (1.821–3.707)	0.000	1.691 (1.161–2.464)	0.006
EuroSCORE II	1.050 (1.039–1.061)	0.000	0.997 (0.977–1.017)	0.733
Previous stroke	1.210 (0.614–2.366)	0.577		
MI ≥ 90 days	1.758 (1.288–2.401)	0.000	1.408 (0.992–1.998)	0.055
Previous cardiac surgery	1.430 (0.732–2.797)	0.295		
Critical preoperative state	1.098 (0.540–2.232)	0.795		
Emergency procedure	1.158 (0.711–1.885)	0.566		
Concomitant surgery	2.045 (1.525–2.742)	0.000	1.244 (0.863–1.793)	0.241
Aorta surgery	1.754 (1.034–2.976)	0.037	2.473 (1.353–54.624)	0.005
Venous grafts only	2.989 (2.204–4.054)	0.000	1.467 (1.009–2.132)	0.034

LVEF = Left ventricular ejection fraction; PAD = peripheral arterial disease; CLD = chronic lung disease; EuroSCORE = European System for Cardiac Operative Risk Evaluation; MI = myocardial infarction.

**Table 4:** Cox-Proportional-Hazard-Analysis for DD patients.

	Univariate		Multivariate	
	Hazard Ratio (95% CI)	*p*-Value	Hazard Ratio (95% CI)	*p*-Value
Gender (male)	0.621 (0.340–1.134)	0.121		
Age	1.044 (1.015–1.074)	0.003	1.043 (1.013–1.075)	0.005
LVEF	0.988 (0.971–1.006)	0.201		
Hypertension	1.028 (0.488–2.163)	0.943		
Diabetes	1.731 (1.039–2.884)	0.035	1.792 (1.045–3.074)	0.034
PAD	1.272 (0.767–2.109)	0.350		
CLD	1.117 (0.604–2.064)	0.724		
EuroSCORE II	1.032 (1.010–1.055)	0.005	1.011 (0.984–1.040)	0.420
Previous stroke	1.260 (0.456–3.482)	0.657		
MI ≥ 90 days	1.871 (1.117–3.132)	0.017	1.549 (0.901–2.662)	0.113
Previous cardiac surgery	0.741 (0.265–2.070)	0.568		
Critical preoperative state	7.622 (2.184–26.96)	0.001	9.723 (2.027–46.643)	0.004
Emergency procedure	0.953 (0.381–2.381)	0.918		
Concomitant surgery	1.618 (0.979–2.676)	0.061		
Aorta surgery	1.652 (0.661–4.130)	0.283		
Venous grafts only	1.146 (0.691–1.899)	0.597		

LVEF = left ventricular ejection fraction; PAD = peripheral arterial disease; CLD = chronic lung disease; EuroSCORE = European System for Cardiac Operative Risk Evaluation; MI = myocardial infarction.

## Data Availability

The datasets generated and analyzed during the current study are available from the corresponding author on reasonable request.
